# Clinical Outcomes after Surgical Aortic Valve Replacement in 681 Octogenarians: A Single-Center Real-World Experience Comparing the Old Patients with the Very Old Patients

**DOI:** 10.3390/geriatrics9020044

**Published:** 2024-04-01

**Authors:** Wilhelm Mistiaen, Ivo Deblier, Karl Dossche, Anthony Vanermen

**Affiliations:** 1 Faculty of Medicine and Health Sciences, University of Antwerp, 2610 Antwerp, Belgium; 2 Department of Cardiovascular Surgery, ZNA Middelheim Hospital, 2020 Antwerp, Belgium; ivo.deblier@zna.be (I.D.); karl.dossche@zna.be (K.D.); anthony.vanermen@zna.be (A.V.)

**Keywords:** surgical aortic valve replacement, octogenarians, mortality, predictors

## Abstract

Aortic valve disease is a lethal condition, once it becomes symptomatic. Surgical aortic valve replacement (SAVR) has, for a long time, been the only treatment option. In patients aged 85 and older, the consequences of SAVR have rarely been investigated. A total of 681 octogenarian patients were subdivided into a group with patients between 80 and 84 years (*n* = 527) and a group with patients aged 85 or older (*n* = 154). For each group, the temporal referral pattern, preoperative comorbid profile, operative data, postoperative need for resources, and adverse postoperative events including 30-day mortality and long-term survival were determined using the chi-squared test, Student’s *t*-test, and log-rank test. For both age groups, the predictors for mortality were identified using a logistic regression analysis. In the oldest patient group, there were significantly more prior episodes of heart failure (75/154 vs. 148/527) and a greater need for urgent SAVR (45/150 vs. 109/515). The operative data and the need for postoperative resources were comparable, but the 30-day mortality was almost twice as high (24/154 vs. 45/527). The need for urgent SAVR was twice as high in the oldest group (odds ratio of 3.12 vs. 6.64). A logistic regression analysis for all 681 patients showed that age over 85 ranked fourth of six predictors for 30-day mortality. Five-year survival was favorable for both groups (67.8 ± 2.1% vs. 60.0 ± 4.3%). A Cox proportional hazard analysis failed to identify an age over 85 as a predictor for long-term mortality. Aortic valve disease and its effect on the left ventricle seemed to be more advanced in the highest age group. The mortality rate was almost double the need for urgent SAVR. This can be avoided by obtaining an earlier referral.

## 1. Introduction

Stenotic and symptomatic calcified aortic valve degeneration (CAVD) is one of the most common age-related cardiovascular conditions in the elderly [1]. Increased premature mortality was documented for all degrees of severity of CAVD in patients aged 65 years or older [2]. While the life expectancy of an 80-year-old person in Western countries is 7–8 years, survival of patients with CAVD after the first occurrence of heart failure was only 2 years [3]. Nevertheless, this condition has been left untreated in the past in a large proportion of patients because of old age or left ventricular or neurologic dysfunction [4]. However, earlier results of surgical aortic valve replacement (SAVR) in octogenarians were rather favorable with a 5-year survival of about 45%. Compared to the need for an urgent SAVR, age above 80 only had half of the impact on early and long-term postoperative outcomes [5]. Furthermore, mortality and postoperative complications decreased over time for all age classes [6]. With the introduction of TAVI, the hospitalization of patients over 85 years increased, with good outcomes for those who underwent valve replacement. A large proportion was still left untreated, however [7,8]. At the same time, the number of octogenarians referred for SAVR also increased [1]. Initially, a high bar was set for the reimbursement of TAVI in Belgium [9]. For this reason, older patients with symptomatic CAVD were mostly referred for SAVR. This was a continuation of a trend that was already observed before the introduction of TAVI in 2008 [10]. There was only a sizable increase in the TAVI volume after 2017, but there were no increases in the age and risk scores [11]. The results of the SAVRs in patients over 80 were well documented, but scarce data exist concerning patients aged 85 and older. The current research questions are as follows: What are the differences between octogenarian patients who are older and younger than 85 years with respect to their preoperative profiles, operative characteristics, need for resources, early postoperative outcomes (mainly mortality), and long-term survival? What are the predictors for these outcomes in both age groups separately? Can an age above 85 be identified as a predictor of early or long-term mortality for octogenarian groups as a whole?

## 2. Materials and Methods

This was a single-center retrospective observational study. Between 1987 and 2017, SAVR was performed in 681 consecutive octogenarian patients who received a biologic heart valve (BHV) in a general teaching hospital. Exclusion criteria were heart valve prosthesis in another position or mechanical valve prosthesis in any position. Patients who underwent associated procedures such as coronary artery bypass grafting (CABG), mitral or tricuspid valve repair, procedure on the ascending aorta, maze procedure, or septal myectomy were also included. A number of preoperative parameters were taken into account. Urgent SAVR was defined as the need for surgery at the index admission, when the diagnosis of CAVD was made. A need for emergency SAVR was defined as the need for surgery within 24 h in order to survive. Acute myocardial infarction (AMI) was documented on ECG or biochemically, and it was labeled as recent if it had occurred less than 3 weeks before SAVR. Chronic kidney dysfunction was defined as a plasma creatinine concentration of over 1.3 mg%, while postoperative acute kidney injury was defined with an increase in plasma creatinine of over 0.3 mg% [12,13]. Chronic pulmonary dysfunction was defined as a forced expiratory volume at 1 s (FEV1) of less than 80% of the predicted value, or the use of chronic bronchopulmonary medication. Diabetes was defined as a fasting plasma glucose level of 125 mg% or more or the use of any antidiabetic treatment. Conduction defects and atrial fibrillation of all types were documented on ECG, while the severity of valve disease (mean and peak gradients, aortic valve area, AVA) was recorded using echocardiography. Coronary artery disease was documented using angiography. The Euroscore II was determined with an online calculator in the last 496 patients. For the first 185 patients, this was not possible, since pulmonary artery pressure was not routinely measured. Patients between 80 and 84 years were compared to patients aged 85 years and older. Both age groups were compared according to their preoperative and operative factors profile, operative data such as associated procedures, cardiopulmonary bypass times, cross-clamp times, and associated procedures as listed. A comparison was also made for the need for resources (blood products, duration of mechanical ventilation, length of stay or LOS in an intensive care unit, postoperative LOS, need for renal replacement therapy, and need for reintervention) and for outcomes such as endocarditis, thromboembolism, bleeding, low cardiac output syndrome, new onset or progression of pre-existing conduction defect, new onset or recurrent atrial fibrillation, acute renal injury, pulmonary complication, delirium with agitation, 30-day mortality, and long-term survival. A two-tailed Pearson chi-squared analysis was used to assess the distribution of categorical preoperative and operative variables and the outcome across both age groups. For continuous variables, Student’s *t*-test was used for independent samples. To identify the predictors and their strengths for hospital mortality, a logistic regression analysis was conducted separately for each age class. To identify the effect of age class on long-term survival, a log-rank test was used, with a Kaplan–Meier plot (SPSS version 29). As an alternative to a propensity score match analysis, a logistic regression was performed for the whole patient group in order to identify independent predictors, especially with respect to the age class. For long-term mortality, a Cox proportional hazard analysis was performed. This study was approved by the ZNA Ethical Committee under protocol No. 2656.

## 3. Results

### 3.1. Temporal Reference Pattern, Preoperative Patient Profile, and Operative Data 

**Figure 1:**
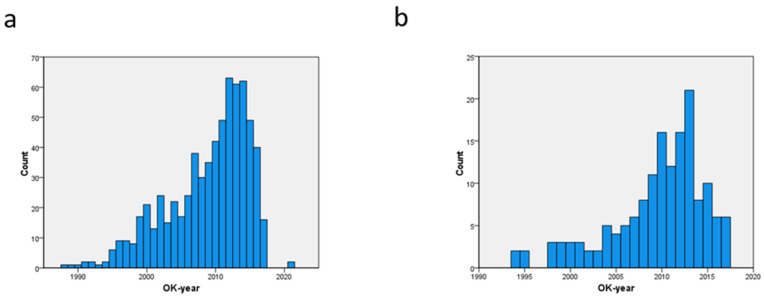
Temporal trends of referral of patients between 80–84 years (**a**) and over 85 years (**b**).

There were 154 patients aged 85 years or older. The preoperative and operative differences with their 527 younger counterparts are displayed in Table 1 and ranked according the *p*-value. The temporal referral pattern is shown in Figure 1a,b. Even after the introduction of TAVI in 2008, the numbers were rising until 2015. Of the 154 patients over 85 years, 47 of these were referred for SAVR before the introduction of TAVI, and 107 patients were referred thereafter. For the 527 patients aged between 80 and 84, these referral numbers were 214 and 313, respectively. The most important cardiac factors present in the oldest group were a prior episode of congestive heart failure and a need for urgent SAVR (i.e., at index admission). However, the need for emergent SAVR (i.e., within 24 h after admission) was not different between age groups (32/527 or 6.1% in the younger group vs. 11/154 or 7.1% in the older group). As can be expected, Euroscore > 8% was also significantly more present in patients aged 85 years or more. Hematologic malignancies were more present in the oldest patient group, but this was not true for malignancies as a whole or for other types of cancer. The oldest patient group did not show significantly more conduction defects or previously implanted permanent pacemakers, atrial fibrillation types, or coronary or any extracardiac arteriopathy. This was also the case for previous cardiovascular interventions (prior PCI, SAVR, or carotid endarterectomy). There were only two patients aged over 85 with endocarditis, of which one was active. No patients of 85 years or older underwent chronic dialysis. The aortic valve area in the older age group was borderline lower (37.2 ± 9.3% vs. 40.4 ± 12.9 mm²) with *p* = 0.059. This observation was associated with a non-significantly higher mean transvalvular gradient (48.0 ± 15.5 mm Hg vs. 46.2 ± 15.3 mm Hg), with *p* = 0.391. Left ventricular ejection fraction was also not significantly different between both age groups (59.1 ± 15.9% vs. 61.6 ± 15.8%) with *p* = 0.166. None of the patients aged over 85 years underwent concomitant carotid endarterectomy. There were 520 patients with a one-vessel disease, 404 patients had a two-vessel disease, and 401 patients had a three-vessel disease without a left main stem involvement. Sixty patients had a left main stem involvement without major lesions in the right coronary artery. In 199 patients, there was an involvement of the left main stem, combined with a severe right coronary artery lesion. The severity of coronary artery disease between the age groups was not significantly different (*p* = 0.235). Cross-clamp time (63.2 ± 20.5 min vs. 67.1 ± 20.3 min) and bypass time (118.7 ± 45.5 min vs. 123.2 ± 40.9 min) were shorter in the older patient group, but this difference was also not significant. The number of bypasses was comparable: 1.6 ± 1.5 vs. 1.5 ± 1.5. There was borderline significantly more partial sternotomy performed in the oldest patient group. Patients aged over 85 years were significantly in a higher risk category with Euroscore II above 8%, but the mean Euroscore II did not differ significantly (9.8 ± 8.8 vs. 8.5 ± 9.6%) with *p* = 0.192. This could related to the finding that the older group was not significantly less present in the low-risk (<4%) or mid-risk (4–8%) group.

### 3.2. Postoperative Adverse Events and Need for Resources

Table 2 shows the effect of the age class on the need for resources and on the postoperative adverse outcome. Need for plasma derivatives, mortality, and pulmonary complications were significantly more observed in patients of 85 years and older. A need for at least four units of packed cells, a prolonged postoperative LOS, and the occurrence of acute renal injury or atrial fibrillation was high for both age groups, but there was no significant difference. LOS in the ICU (4.2 ± 7.0 days vs. 3.6 ± 7/6 days), postoperative hospital stay (11.6 ± 7.5 days vs. 11.4 ± 10.1 days), and duration of mechanical ventilation (19.3 ± 46.3 h vs. 18.5 ± 54.0 h) were not significantly prolonged in the older group (*p* > 0.4 for all). Only one case of postoperative endocarditis was observed in patients 85 or older, and two cases were documented in the younger group. There were no cases of myocardial damage in patients 85 and older, but there were nine cases in the younger age group. All lab values (lowest hematocrit < 25%, highest plasma glucose > 160 mg%, and lowest pO_2_ < 80 mm Hg) were not significantly different.

### 3.3. Independent Predictors for Short-Term Outcome 

Table 3 compares the independent predictors for 30-day mortality in both patient age groups. Both patient age groups tolerated postoperative acute renal injury equally poorly. However, the effect of the need for urgent SAVR on mortality in patients over 85 years was far stronger compared to the younger patient age group. Reintervention was the most clinically relevant predictor in the group of 80 to 84 years, but this effect was less significant due to the low numbers (5 vs. 14 cases for the older and younger age groups, respectively). Due to the lower number in the oldest patient group, only two predictors were identified in order to prevent overfitting of the statistical model. The need for reintervention and incompleteness of revascularization had no significant effect in the older group. 

Table 4 shows the six independent predictors of 30-day mortality for the whole group of 681 octogenarian patients. The need for emergent SAVR was the strongest predictor and was almost twice as strong compared to the age factor (>85 years).

### 3.4. Long-Term Outcome

**Figure 2:**
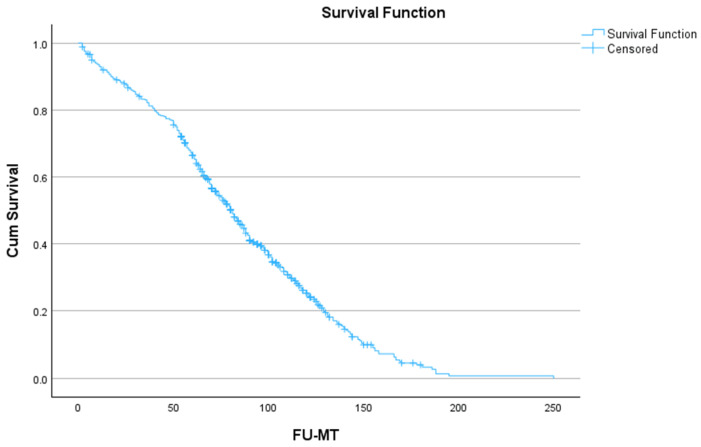
Long-term outcome after surgery for all octogenarian patients.

The difference in long-term outcome (Figure 2 and Table 5) between age groups was significant (*p* = 0.050), although this difference is limited: the mean survival time in the younger group was 86.1 (81.6–90.7) months; in the oldest, it was 77.7 (68.5–86.9) months. From the data in Table 5, it can be derived that the divergence of more than 5% between the age groups occurred only after 5 years of follow-up.

Figure 2 shows the long-term outcome for the whole group of octogenarians.

**Figure 3:**
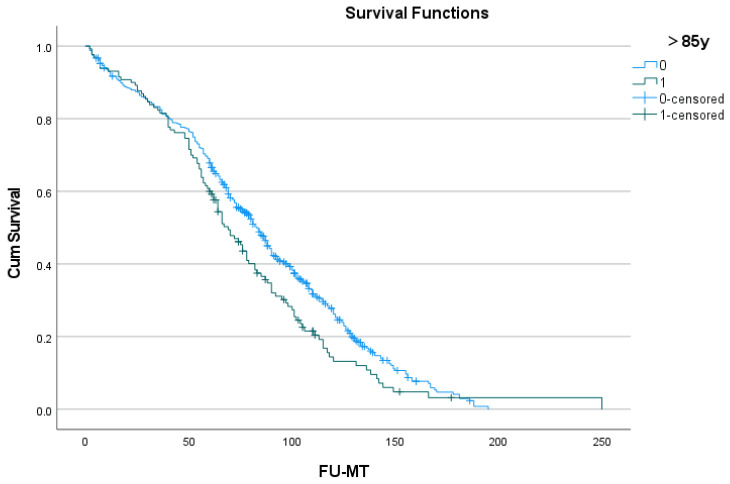
Long-term outcome after surgery in patients of 80–84 years (blue) and 85 years or older (green).

Figure 3 shows the long-term outcome for patients of 80–84 years vs. 85 years and older.

For the whole group of 681 octogenarian patients, nine independent predictors for long-term mortality were identified (Table 6). Six of these predictors were of a preoperative nature. Postoperative cardiac events and the need for emergent SAVR had the strongest effect. Age above 85 years was not identified as an independent predictor.

## 4. Discussion

The referral of octogenarians for SAVR in our institution has increased over time, even after the introduction of TAVI in 2008. This finding also applied to patients 85 years and older. This observation could be due to the reimbursement policy. The differences in preoperative profile between patients of 80–84 years and 85 years or older were limited to congestive heart failure, a need for urgent SAVR, and prior hematological malignancies. The first two factors indicated that the effect of aortic valve disease on the left ventricle was more advanced in very elderly patients. These patients had also a higher risk of a Euroscore II of over 8%, although the difference in mean Euroscore was only 1.3%, which was not significantly higher. The cross-clamp time and bypass time were shorter in the older age group, but these differences were also not significant. In the older age group, an approach through partial sternotomy was performed more often, but none of the patients of this patient age group underwent concomitant carotid endarterectomy. The only significant difference in resources was the need for plasma derivatives. The oldest patient group had a significantly higher rate of pulmonary complications such as atelectasis and pneumonia, as well as 30-day mortality.

The current results indicated that postoperative acute renal failure, as the most important and significant predictor, was poorly tolerated by both patient age groups equally. Preoperative chronic kidney disease, which has been identified earlier as the single most important determinant for this postoperative adverse event [12], was also almost equally present in both age groups. Perioperative measures should be taken to protect the kidneys, especially when chronic renal dysfunction is documented. The second predictor, the need for urgent SAVR (i.e., surgery needed at index admission) was more present in patients of 85 years and older. It predicted the 30-day mortality with an odds ratio over 6, while this was just over 3 in the younger patient age group. This should be considered as more relevant since the development of this predictor might be prevented by early referral. The need for reintervention was more clinically relevant with an odds ratio of 8 in the younger age group. This predictor, however, was less significant due to lower numbers and could not be found in the higher-age patient group. For incomplete revascularization, a comparable observation was made.

In order to account for the effect of age itself on the short-term outcome of SAVR, a multivariate analysis was performed for the octogenarian group as a whole. Six predictors were identified, of which age over 85 was ranked fourth. The need for emergent SAVR (i.e., within 24 h of admission) was clinically the most relevant and much more significant. The four remaining predictors (coronary artery disease, atrial fibrillation, renal or pulmonary disease) had a strength comparable to age over 85 years. This observation confirmed an earlier report concerning the devastating effect of the pressure overload on an aging left ventricle and its inability to maintain adequate circulation [5]. There was a significant difference (*p* = 0.050) in long-term survival between both age groups, but age above 85 was not identified as a predictor in a Cox proportional hazard analysis. Of the nine predictors that were identified, six were preoperative in nature, and three were postoperative adverse events.

There are only a few reports that focus on the outcome after SAVR in patients of 85 and older, and sample sizes are usually small. This age category falls between the categories of octogenarians and nonagenarians. In one very small Japanese series of 29 patients aged above 85 years, no mortality was observed, and the introduction of the TAVI procedure had no clear effect on other outcomes. As in our series, a temporal increase in the referral of octogenarians for SAVR was observed. There were only a few differences between patients who underwent isolated SAVR and SAVR with CABG. The Euroscore II for both groups was below 6%, which makes this Japanese series an outlier. Moreover, the reported survival in the patient group who underwent isolated SAVR was unusually high [1]. The rate of major postoperative complications was 13% before the introduction of TAVI, but this increased to 23% afterward. The mean LOS in the ICU increased from 3 to 4 days, while the mean postoperative LOS increased from 18 to 27 days. The risk scores did not change, however. Moreover, the number of patients was too low to reach a level of significance [13]. A second, larger Japanese series studied the effect of age above 85 years in 50 patients from a series totaling 161 octogenarians who underwent SAVR with or without CABG. Hospital mortality for all patients was 4.3%, which was low. Age over 85 was not identified as a predictor for any adverse postoperative event, but a Euroscore II of over 10% and extracardiac arteriopathy were predictive. The overall survival rate at 1 and 4 years postoperatively was 91.0% and 46.9% [14], somewhat lower compared to the current results. No multivariate analysis was performed. The effect of age class (younger than 80 years vs. 80 to 84 years vs. 85 years or older) on postoperative outcome was studied after SAVR with or without CABG in a much larger group [15]. The 30-day mortality in these three age groups after isolated SAVR was 3.7%, 6.7%, and 11.7%, respectively, while the mean long-term survival was 11.5, 6.8, and 6.2 years, respectively. Among patients undergoing SAVR with concomitant CABG, the 30-day mortality also increased significantly among these age groups, with 6.2%, 9.4%, and 8.5%, respectively, while the mean survival time was 9.4, 6.8, and 7.1 years. Plotting the survival curves showed a large difference between patients younger than 80 and older than 80 but not between the patients aged between 80 and 84 vs. patients older than 85 years. The focus of this series was on the association of CABG in the different age groups and not on other potential predictors for poor outcomes [15].

Some series reported the results of SAVR in nonagenarians, a population that comes close to the current one, but these patient series were usually small and dealt with cardiac surgery of all types. In one series, it was clear that the involvement of the aortic valve in the disease process was associated with a higher rate of congestive heart failure, which pointed towards the importance of pressure overload on the aged left ventricle. The mortality in valve-only (12.8%) and valve plus CABG (18.9%) was higher compared to CABG-only (8.8%) and in range with the currently observed results. These differences, however, were not significant. This was probably due to low patient numbers. Cardiac surgery could be safely performed in this age group, but long-term survival in nonagenarians was lower compared to octogenarians and septuagenarians, which in itself is not surprising. A four-year survival of nonagenarians of about 50% should be considered as a good result. The difference in survival between valve-only and valve with CABG was only visible between 2 and 4 years follow-up and disappeared at 5 years. An increment in age of one year in the nonagenarian group was the most significant predictor for survival, but its impact in terms of hazard ratio was lower than the impact of prior cardiac surgery or of a prior CVA [16]. However, in another series, risk scores such as STS-PROM underestimated the operative risk in nonagenarians undergoing valve operations. Compared to younger counterparts, preoperative comorbid conditions and especially congestive heart failure were more present in the patients aged 90 or more. Almost all types of postoperative adverse events were also significantly more present in this age group. Early mortality in nonagenarians was 18.0% vs. 2.6% in the younger group. However, as the surgical volume increased over time, the mortality rate also decreased in these very old patients [17]. A comparison between octogenarians and nonagenarians undergoing cardiac surgery showed a higher rate of valve surgery in the older group. The need for urgent surgery, cross-clamp and cardiopulmonary bypass time, hospital complications, and mortality rates were not different. However, both age groups had a high portion of an urgent status of 25–27%. Only chronic kidney dysfunction and atrial fibrillation were identified as predictors for mid-term outcomes [18].

Octogenarian patient groups are larger compared to nonagenarian series and usually limited to SAVR, with or without an additional procedure. Our current results showed a higher mortality in the patient age group of 85 years and older, which was comparable with the postoperative results in octogenarians published in 2001. Mortality in this earlier series was 5% after SAVR and 15% after SAVR with concomitant CABG [3]. The effect of age on outcome was clearly demonstrated in a more recent series. Age above 80 was an independent predictor for 30-day mortality after SAVR. The raw data showed a mortality rate of 21.6% in older vs. 5.8% in younger patients [19]. These results could be compared with another series with an operative mortality rate of 9% for SAVR and 24% for SAVR with CABG. Although age was identified as the strongest predictor for a prolonged hospital stay, it was not identified as a predictor for operative mortality. However, age had the most significant effect on long-term survival, followed by a previous AMI, a need for an urgent procedure, and a prolonged duration of ICU stay [20]. In another large series, operative mortality in octogenarians was 5.5% for SAVR and 11.5% for SAVR with CABG. The predictors of operative mortality were NYHA functional class IV, age, atrial fibrillation, and SAVR with an associated procedure [21]. In the most recently published larger series of octogenarian patients, mortality was much lower: this was only 2.2% for isolated SAVR and 3.2% for combined procedures. Survival rates at 1 y (90%), 5 y (66%), 10 y (31%), and 15 y (14%) were very close to the current patient group aged between 80 and 84 years, and somewhat better compared to the patients of 85 years and older. Age, gender, high NYHA class, peripheral artery disease, myocardial infarction, earlier era, and cross-clamp and cardiopulmonary bypass times were independent predictors for long-term mortality after SAVR with or without other procedures [22]. In one remarkable study, octogenarians who were referred for TAVI by a heart team but were subsequently redirected for SAVR were compared to octogenarians referred for SAVR without such screening by a team. After propensity score matching, both groups were largely comparable, although patients screened by the heart team had more congestive heart failure and pulmonary hypertension. Nevertheless, mortality was much lower in the patients screened by the heart team, with a bootstrapped difference of 6% (2.2–9.8%). The patient numbers of both groups were low (*n* = 76), and the results should therefore be treated with caution. Nevertheless, this indicated that an assessment by a heart team before intervention was associated with lower in-hospital mortality and complication rates [23]. It also indicated that SAVR is still an acceptable option in selected elderly patients.

## 5. Conclusions

The most significant and clinically relevant differences in preoperative profile between both age groups indicate a more advanced disease and a higher need for urgent SAVR in the patient group of 85 years and older. The operative characteristics as well as the need for postoperative resources were for the most part comparable. The thirty-day mortality rate was almost double in the group aged over 85. The need for an urgent SAVR as a predictor for 30-day mortality was the most clinically relevant finding: this was twice as high in the oldest group. This indicated that this patient age group tolerated the progress of aortic valve disease and its impact on the capacity of the left ventricle to generate adequate circulation very poorly. Long-term survival was favorable for both age groups but was 9 months shorter in the oldest group. SAVR should not be discouraged for the sake of age alone. Since the need for an urgent SAVR was significantly higher in the older age class, hesitancy to perform SAVR because of a potential poor outcome could be a self-fulfilling prophecy. However, mortality remained high, and TAVI should be considered in this age group. If comparable predictors are also identified in patients undergoing TAVI, valve replacement by any method should not be postponed once valve-related symptoms appear in these very elderly patients.

## 6. Limitations

This was a retrospective observational study with all its inherent limitations. However, patients were consecutively included, thereby limiting sources of bias. In a large time frame, the operative technique and perioperative care have improved. These factors were not taken into account. In the absence of a propensity score match analysis, a multivariate logistic regression analysis was performed for 30-day mortality for the whole octogenarian group. This analysis provides information on the effect of age on outcome. For long-term mortality, a comparable analysis was performed using a Cox proportional hazard analysis. The need for an urgent and emergent SAVR was not always clearly defined in the referenced series, which could make comparison with their outcomes sometimes more difficult. The quality of life of these patients is more important than the prolongation of life. However, the available data for the current population were insufficient for proper analysis since quality of life was not routinely screened during the follow-up and many patients were admitted to skilled nursing facilities.

## Figures and Tables

**Table 1:** The effect of age class on the distribution of preoperative and operative factors.

Preop and Operative Factors	>85 y (%)	80–84 y (%)	*p*
Preoperative factors			
Congestive heart failure	75/154 (48.7)	148/527 (28.0)	0.001
Need for urgent SAVR	45/150 (30.0)	109/515 (21.2)	0.024
Euroscore II > 8%	51/124 (41.1)	113/371 (30.5)	0.029
Hematological malignancy	7/131 (5.3)	9/446 (2.0)	0.042
BMI > 30 kg/m^2^	17/122 (13.9)	79/365 (21.6)	0.064
Conduction defect (all types)	68/154 (44.2)	195/526 (37.1)	0.112
Diabetes mellitus	36/154 (23.4)	94/527 (17.8)	0.121
Coronary artery disease	98/154 (63.9)	370/527 (70.2)	0.122
NYHA III/IV	94/110 (85.5)	313/396 (79.0)	0.134
Arterial hypertension	108/154 (70.1)	395/523 (75.5)	0.178
Prior CABG	19/154 (12.3)	46/527 (8.7)	0.180
Plasma creatinine > 1.3 mg%	43/154 (27.9)	130/527 (24.7)	0.414
Myocardial infarction	30/154 (19.5)	89/525 (17.0)	0.468
Male gender	79/154 (51.3)	278/528 (52.7)	0.567
Complex ventricular arrhythmia	18/154 (11.7)	55/527 (10.4)	0.659
Peripheral artery disease (all)	57/146 (39.0)	200/488 (41.9)	0.675
Atrial fibrillation (all types)	47/154 (30.5)	169/527 (32.1)	0.920
All neurologic ischemic events	26/154 (16.9)	83/527 (15.7)	0.729
Pulmonary artery hypertension	45/124 (36.3)	134/387 (34.6)	0.735
Left ventricular hypertrophy	127/140 (90.7)	427/586 (89.9)	0.781
Left ventricular ejection fraction < 50%	23/112 (20.5)	75/375 (20.0)	0.901
FEV1 < 80% predicted value	42/146 (28.8)	147/507 (29.0)	0.958
Operative factors			
Partial sternotomy	16/14 (10.8)	31/511 (6.1)	0.048
Cross-clamp time > 60 min	66/112 (54.1)	248/397 (62.5)	0.098
Concomitant CABG	91/154 (59.1)	349/527 (66.1)	0.110
Procedure on the ascending aorta	5/154 (3.2)	31/527 (5.9)	0.197
Perceval valve prosthesis^®^	10/154 (6.5)	23/527 (4.4)	0.277
Incomplete revascularization	22/149 (14.8)	66/513 (12.9)	0.548
Smallest valve size	5/153 (3.3)	21/536 (4.0)	0.681
Mitral valve repair	7/154 (4.5)	22/527 (4.2)	0.839
Cardiopulmonary bypass time > 120 min	59/123 (42.8)	198/466 (42.5)	0.956

BMI: body mass index; CABG: coronary artery bypass graft; FEV1: forced expiratory volume at 1 s; NYHA: New York Heart Association; y: years.

**Table 2:** The effect of age class on postoperative adverse events and need for resources.

Postoperative Events and Resources	>85 y (%)	80–84 y (%)	*p*
Need for resources			
Plasma derivatives	46/121 (38.0)	100/369 (27.1)	0.023
Length of stay in intensive care unit > 1 day	64/137 (46.7)	163/430 (37.9)	0.067
Mechanical ventilation > 8 h	62/124 (50.0)	156/367 (42.5)	0.147
Renal replacement therapy	13/154 (8.4)	28/526 (5.3)	0.153
Thrombocyte concentrate	20/121 (16.5)	50/369 (13.6)	0.416
Postoperative length of stay > 8 days	74/147 (50.3)	230/494 (46.6)	0.420
Permanent pacemaker implant	7/154 (4.5)	18/527 (3.4)	0.509
>4 units packed cells	35/121 (28.9)	97/369 (26.3)	0.570
Reintervention	5/154 (3.2)	14/528 (2.6)	0.693
Adverse events			
30-day mortality	24/154 (15.6)	45/527 (8.5)	0.011
Pulmonary complications	36/154 (23.4)	83/527 (15.7)	0.028
Thromboembolic event	10/154 (6.5)	17/527 (3.2)	0.068
Bleeding event	18/154 (11.7)	40/527 (7.6)	0.109
Low thrombocytes	16/123 (13.0)	65/371 (17.5)	0.371
New or progressing conduction defect	29/154 (18.8)	115/627 (21.8)	0.424
Acute renal injury	53/154 (34.4)	164/527 (31.1)	0.440
Low cardiac output syndrome	20/153 (13.1)	60/527 (11.4)	0.569
Recurrent or new-onset atrial fibrillation	65/154 (42.2)	209/527 (39.7)	0.570
Pneumothorax, prolonged chest drain	36/154 (23.4)	114/527 (21.6)	0.646
Delirium with agitation	23/143 (16.1)	84/490 (17.1)	0.766
Ventricular arrhythmias	7/154 (4.5)	23/527 (4.4)	0.923
Length of stay in days	4.2 ± 7.0	3.6 ± 7.6	0.402
Plasma creatinine (mg%)	1.61 ± 0.97	1.70 ± 1.03	0.420
Left atrial pressure (cm H_2_O)	18.8 ± 4.9	18.4 ± 4.8	0.553
Mean transvalvular gradient (mm Hg)	11.6 ± 5.0	11.0 ± 5.4	0.577
Postoperative length of stay (days)	11.6 ± 7.5	11.4 ± 10.1	0.753
Increase in plasma creatinine (mg%)	0.53 ± 0.80	0.51 ± 0.75	0.821
Units of packed cells	3.4 ± 3.5	3.3 ± 4.1	0.829
Mechanical ventilation duration (hours)	19.3 ± 46.3	18.5 ± 54.0	0.863
Peak transvalvular gradient (mm Hg)	19.1 ± 8.0	19.2 ± 9.4	0.940

cm: centimeter; mm Hg: millimeter mercury; y: years.

**Table 3:** Independent predictors for 30-day mortality for both age groups.

	80 to 84 y		>85 y	
Predictor	OR (95% CI)	*p*	OR (95% CI)	*p*
Acute renal injury	5.69 (2.62–12.33)	<0.001	6.65 (2.18–19.65)	<0.001
Urgent SAVR	3.12 (1.49–6.51)	0.002	6.64 (2.27–19.34)	<0.001
Reintervention	8.01 (2.04–31.46)	0.003	-	-
Incomplete revasc.	2.96 (1.25–7.03)	0.014	-	-

CI: confidence interval; OR: odds ratio; revasc: revascularization; SAVR: surgical aortic valve replacement; y: years.

**Table 4:** Multivariate analysis for identification of predictors for 30-day mortality in the whole patient group.

Predictor	Odds Ratio	95% CI	*p*
Emergent SAVR	3.87	1.74–8.64	0.001
FEV1 > 80% predicted	2.44	1.39–4.26	0.002
Atrial fibrillation	2.30	1.30–4.06	0.004
Age > 85 y	2.02	1.11–3.66	0.021
Preoperative plasma creatinine > 1.3 mg%	1.94	1.09–3.45	0.025
Coronary artery disease	1.99	1.01–3.89	0.046

FEV1: forced expiratory volume 1 s; SAVR: surgical aortic valve replacement; y: years.

**Table 5:** Long-term data for both patient age groups (*n* = patients at risk); *p* = 0.050.

Time	80–84 y	*n*	<85 y	*n*	All Patients	*n*
1 year	91.7 ± 1.3%	444	93.1 ± 2.2%	120	92.0 ± 1.1	564
5 years	67.8 ± 2.1%	329	60.0 ± 4.3%	78	66.4 ± 1.9	409
10 years	26.2 ± 2.2%	86	13.2 ± 3.4%	11	25.3 ± 2.0%	97

*n*: number; y: years.

**Table 6:** Cox proportional hazard analysis for long-term mortality for the whole patient group.

Predictor	Odds Ratio	95% CI	*p*
Preoperative plasma creatinine < 1.3 mg%	1.40	1.11–1.76	0.005
Preoperative congestive heart failure	1.37	1.09–1.71	0.006
Postoperative low cardiac output syndrome	1.82	1.18–2.83	0.007
Postoperative delirium	1.45	1.09–1.92	0.010
Postoperative ventricular arrhythmia	1.84	1.13–3.00	0.021
Emergent SAVR (<24 h)	1.69	1.08–2.65	0.021
Diabetes mellitus	1.30	1.02–1.66	0.032
Preoperatively treated malignancy	1.30	1.01–1.69	0.046
Preoperative FEV1 < 80% predicted value	1.26	1.00–1.57	0.047

FEV1: forced expiratory volume 1 s; SAVR: surgical aortic valve replacement.

## Data Availability

The data in this manuscript are derived from a multipurpose database, which will be used for further exploration. For this reason, these data cannot be shared.
